# A mouse model for the study of anti-tumor T cell responses in Kras-driven lung adenocarcinoma

**DOI:** 10.1016/j.crmeth.2021.100080

**Published:** 2021-09-16

**Authors:** Brittany Fitzgerald, Kelli A. Connolly, Can Cui, Eric Fagerberg, Dylan L. Mariuzza, Noah I. Hornick, Gena G. Foster, Ivana William, Julie F. Cheung, Nikhil S. Joshi

**Affiliations:** 1 Department of Immunobiology, Yale University School of Medicine, New Haven, CT 06519, USA; 2 Lead contact

## Abstract

Kras-driven lung adenocarcinoma (LUAD) is the most common lung cancer. A significant fraction of patients with Kras-driven LUAD respond to immunotherapy, but mechanistic studies of immune responses against LUAD have been limited because of a lack of immunotherapy-responsive models. We report the development of the immunogenic KP × NINJA (inversion inducible joined neoantigen) (KP-NINJA) LUAD model. This model allows temporal uncoupling of antigen and tumor induction, which allows one to wait until after infection-induced inflammation has subsided to induce neoantigen expression by tumors. Neoantigen expression is restricted to EPCAM+ cells in the lung and expression of neoantigen was more consistent between tumors than when neoantigens were encoded on lentiviruses. Moreover, tumors were infiltrated by tumor-specific CD8 T cells. Finally, LUAD cell lines derived from KP-NINJA mice were immunogenic and responded to immune checkpoint therapy (anti-PD1 and anti-CTLA4), providing means for future studies into the immunobiology of therapeutic responses in LUAD.

## In brief

Fitzgerald et al. develop a mouse model of LUAD that generates an endogenous CD8 T cell response against lung tumors. Tumors from this model respond to immunotherapy, providing a new tool for researchers to use to investigate the processes that underlie immunotherapy responses.

## Graphical abstract

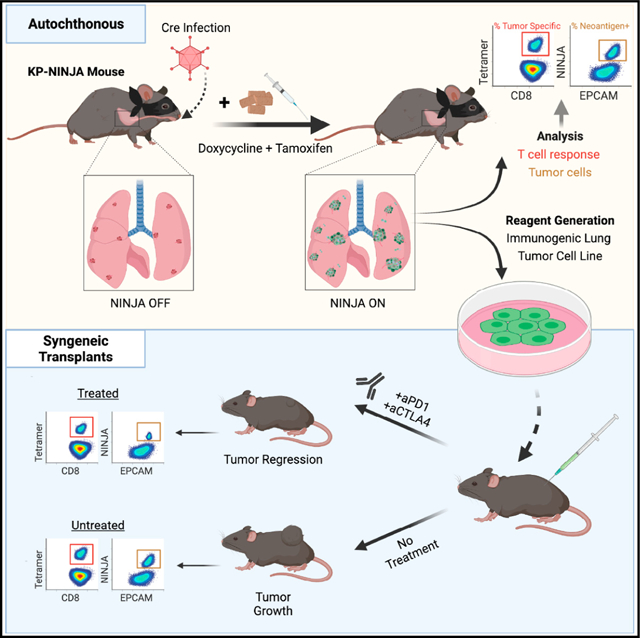

## INTRODUCTION

Breakthroughs in our understanding of cancer biology have proceeded apace with model development in the field, and thus choosing a suitable model for the question under investigation is critical (Gengenbacher et al., 2017; Sanmamed et al., 2016). Transplantable tumor models are widely used and are particularly useful for screening potential new drugs for therapeutic efficacy (Burton and Begg, 1961; DeVita and Chu, 2008; Frederico et al., 2017; Iwai et al., 2002, 2005; Leach et al., 1996; Lee et al., 2002). By contrast, spontaneous or inducible genetically engineered mouse models (GEMMs) of cancer feature physiologically developing tumors, often with relevant genetic alterations for patient pathology (Aguirre et al., 2003; Brinster et al., 1984; Chin et al., 1999; Dankort et al., 2009; Dinulescu et al., 2005; Donehower et al., 1992; DuPage et al., 2009; Fisher et al., 2001; Hakem et al., 1996; Hanahan, 1985; Hingorani et al., 2003a, 2003b, 2005; Jacks et al., 1992; Jackson et al., 2001; Johnson et al., 2001; Pelengaris et al., 1999; Rao et al., 2004; Rose-Hellekant and Sandgren, 2000; Stewart et al., 1984; Xu et al., 1999; Zender et al., 2006). Our understanding of human disease hinges on the quality and prevalence of models that both recapitulate physiology and allow the isolation and manipulation of disease variables.

Non-small-cell lung cancer (NSCLC) is a deadly form of cancer, accounting for >20% of all cancer deaths annually in the US (Siegel et al., 2021). Kras-driven lung adenocarcinoma (LUAD) is the most common form of NSCLC and remains a significant source of cancer mortality despite recent advances in therapy. Targeted therapies have benefited patients with EGFR and Alk mutant lung cancers, but for decades, therapeutic options for patients with Kras mutant lung cancer did not extend beyond chemo/radiotherapy or surgery, contributing to the low survival rates (Fidias and Novello, 2010; Lynch et al., 2004; Molina et al., 2008). With the growing recognition in the 1990s that both lymphocytes and innate immune cells play a critical role in cancer responses (Raez et al., 2005; Smyth et al., 2001) and with the paradigm shifting success of immune checkpoint therapies (Chambers et al., 1999; Hirano et al., 2005; Howlader et al., 2020; Iwai et al., 2002, 2005; Korman et al., 2006), interest in the area of cancer immunology expanded rapidly. Indeed, immune checkpoint therapy is now the FDA-approved first-line treatment for LUAD, and Kras mutant lung tumors are capable of response, although response rates remain low.

For Kras-driven LUAD research, the KP (Kras^lox-stop-lox(lsl)-G12D/+^; p53^flox/flox^) GEMM has been a widely used inducible tumor model that recapitulates the cardinal features of the human disease. This model has enabled the observation of disease progression from the moment of tumor transformation and has provided valuable insight into questions of cancer development (Ferone et al., 2016). KP mice and other existing GEMMs are excellent for studying cancer cell intrinsic changes, but present difficulties for studying immunology, as they contain few neoantigens and elicit poor T cell responses (Blasco et al., 2019; DuPage et al., 2009, 2011; Evans et al., 2016; McFadden et al., 2016; Wang et al., 2017; Westcott et al., 2015). KP mouse tumors also differ from human LUADs because they remain refractory to checkpoint immunotherapy (Adeegbe et al., 2018; Herter-Sprie et al., 2016; Li et al., 2017; Lim et al., 2018; Pfirschke et al., 2016; Van Damme et al., 2021).

The uncertainty regarding immune responses in GEMMs meant that much of lung cancer research, until recently, was conducted using human cell line xenografts in immunocompromised mice (Kellar et al., 2015) or syngeneic mouse lines. One syngeneic mouse lung tumor cell line commonly used for tumor transplant studies into immunocompetent hosts is LLC. Early studies showed that LLC tumors were relatively non-immunogenic (Treves et al., 1976), e.g., they formed tumors with similar penetrance in immunocompetent and irradiated hosts (Van Pel et al., 1979). LLC immunogenicity was altered after *N*-methyl-*N*′-nitro-*N*-nitrosoguanidine mutagenesis, leading to subclones that were rejected in immunocompetent hosts due to cytotoxic lymphocyte functions (Van Pel et al., 1979; Vessiere et al., 1982). However, many of these subclones lost immunogenicity after months in culture, likely due to genomic instability in LLC (Van Pel et al., 1979). LLC derivatives have also been generated that express ovalbumin (LLC-OVA), but neither the Ova+ nor parental cells respond consistently to immune checkpoint therapy (Lim et al., 2018; Van Damme et al., 2021). CMT167 is another mouse LUAD line, and has been shown to respond to anti-PD1 or anti-PD-L1 in C57BL/6J mice (Bullock et al., 2019; Li et al., 2017). CMT167 is a derivative of the metastatic CMT 64 LUAD cell line that was generated >40 years ago from a spontaneous tumor in a C57BL/*lIcrf-a*^*t*^ mouse (Franks et al., 1976; Layton and Franks, 1984), a strain that was established as an independent colony from C57BL/6J in 1940 (Rowlatt et al., 1976). Thus, while CMT167 is certainly useful, it is uncertain to what extent genetic drift accounts for the immunogenicity of this line. Due to the lack of consistent immunotherapy responses in these lines, there is a clear need in the field for an immunogenic mouse LUAD cell line that is also responsive to immune checkpoint therapies.

There has also been copious effort put into generating GEMM models that overcome the lack of immune responses seen in the KP model of LUAD. This centered around efforts to increase autochthonous tumor immunogenicity by programming tumors to express neoantigens, which elicit T cell responses and allow for the investigation of tumor antigen-specific T cell responses. The methods for this include genomically encoded inducible neoantigens, lentiviral/transposon-based delivery, and electroporation (Alonso et al., 2018; Cheung et al., 2008; DuPage et al., 2009, 2011, 2012; Hegde et al., 2020; Radkevich-Brown et al., 2010; Schietinger et al., 2016; Spranger et al., 2016; Van Pel et al., 1979; Wang et al., 2017). Each method has advantages and drawbacks, with the latter often being unintended impacts on the anti-tumor immune response. For example, intratracheal (i.t.) delivery of neoantigen- and Cre-expressing lentiviruses (LVs) in KP mice results in the formation of neoantigen+ lung tumors. LVs are advantageous because the integration of the provirus is obligate for Cre expression by LV-infected lung cells, and Cre expression is required for activation of Kras^G12D^ and elimination of p53, which leads to tumor formation. Thus, all LV-infected tumors are programmed to express neoantigens, which elicits a neoantigen-specific CD8 T cell response (DuPage et al., 2011). Yet lentiviral infection is accompanied by caveats: i.t. lentiviral infection itself is associated with inflammation (Brown et al., 2007), proviruses can be silenced after integration, and LVs transduce non-tumor immune cells that could also elicit “anti-tumor” T cell responses (DuPage et al., 2011). These caveats can confound the interpretations of immune responses in these models. Thus, there is a critical need to develop new models of LUAD for cancer immunology that generate quantifiable and selective anti-tumor immune responses.

We recently published the NINJA (inversion inducible joined neoantigen) mouse model, which allows for *de novo*, highly regulated neoantigen induction *in vivo* (Damo et al., 2020). Unlike previous inducible neoantigen models, NINJA does not have leaky expression of neoantigen in thymocytes or peripheral tissues prior to induction, and thus avoids central and peripheral tolerance of endogenous neoantigen-specific CD8 T cells. The Cre-inducible regulatory elements in the NINJA model were designed to be compatible with the KP model, and to address many of the caveats with lentiviral neoantigen programming. Here, we describe the KP-NINJA LUAD model and demonstrate its strengths for studies of tumor immunology.

## RESULTS

### Optimizing Cre delivery to limit the impacts of inflammatory cytokines and fraction of bystander Cre+ cells in the lung

**Figure 1.**
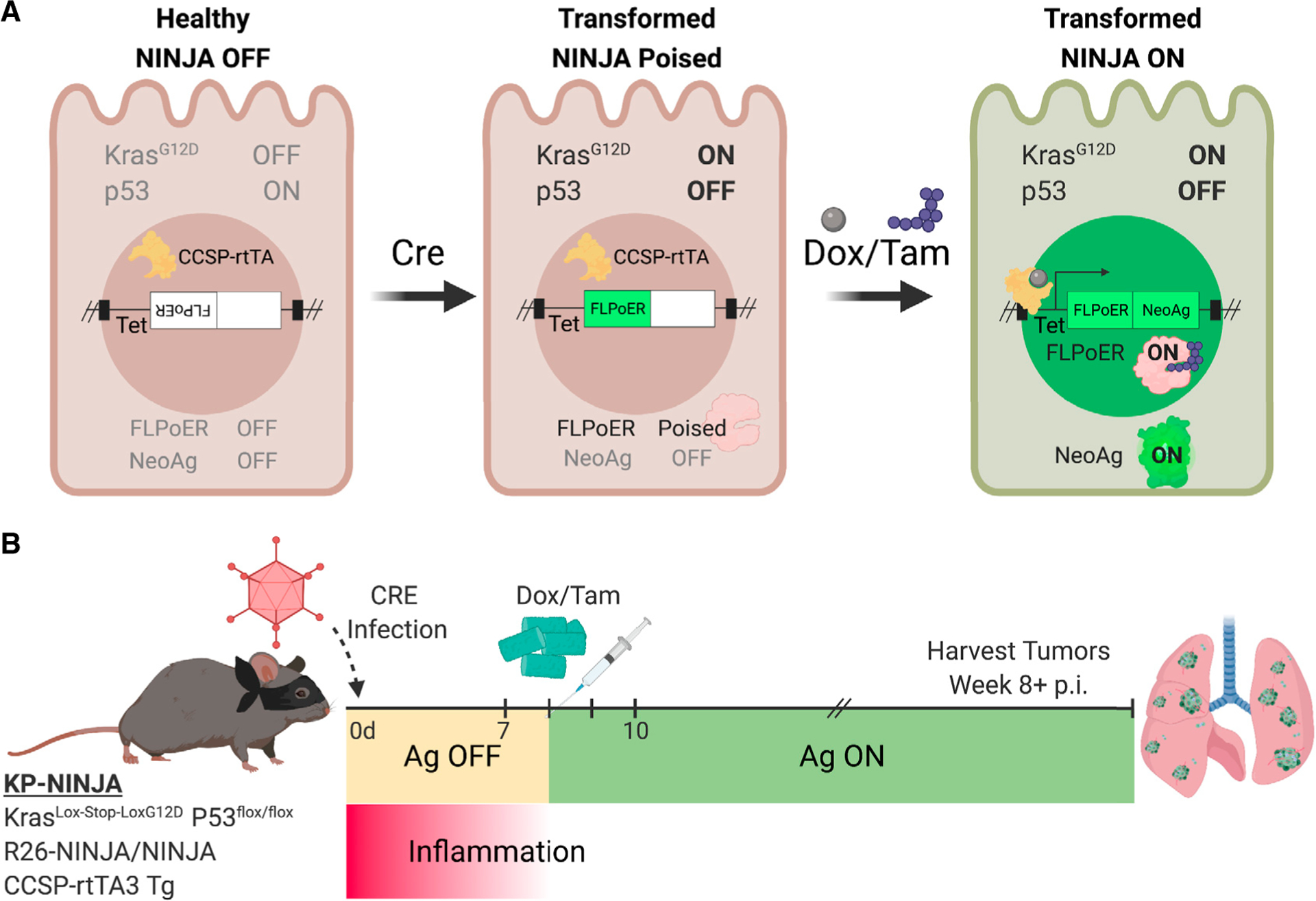
KP-NINJA lung tumor neoantigen induction model (A) Schematic of NINJA and KP allele induction activity. Kras^Lox-Stop-Lox (LSL)G12D^ is activated and p53^flox/flox^ is deleted after Cre recombinase activity, and the FLPoER in NINJA flips to being in-frame for transcription. After doxycycline and tamoxifen (Dox/Tam) administration, Dox binds the lung-specific CCSP-rtTA to allow the Tet-controlled FLPoER promoter to express, and Tam binds FLPoER to allow nuclear entry and recombination and expression of the NINJA neoantigen. (B) KP-NINJA mouse tumor and antigen induction protocol timeline, with Adeno- or Lenti-Cre induction at day 0, followed by antigen induction by Dox/Tam at days 7–10. Inflammation approximation is based on data presented in Table 1.

**Figure 2.**
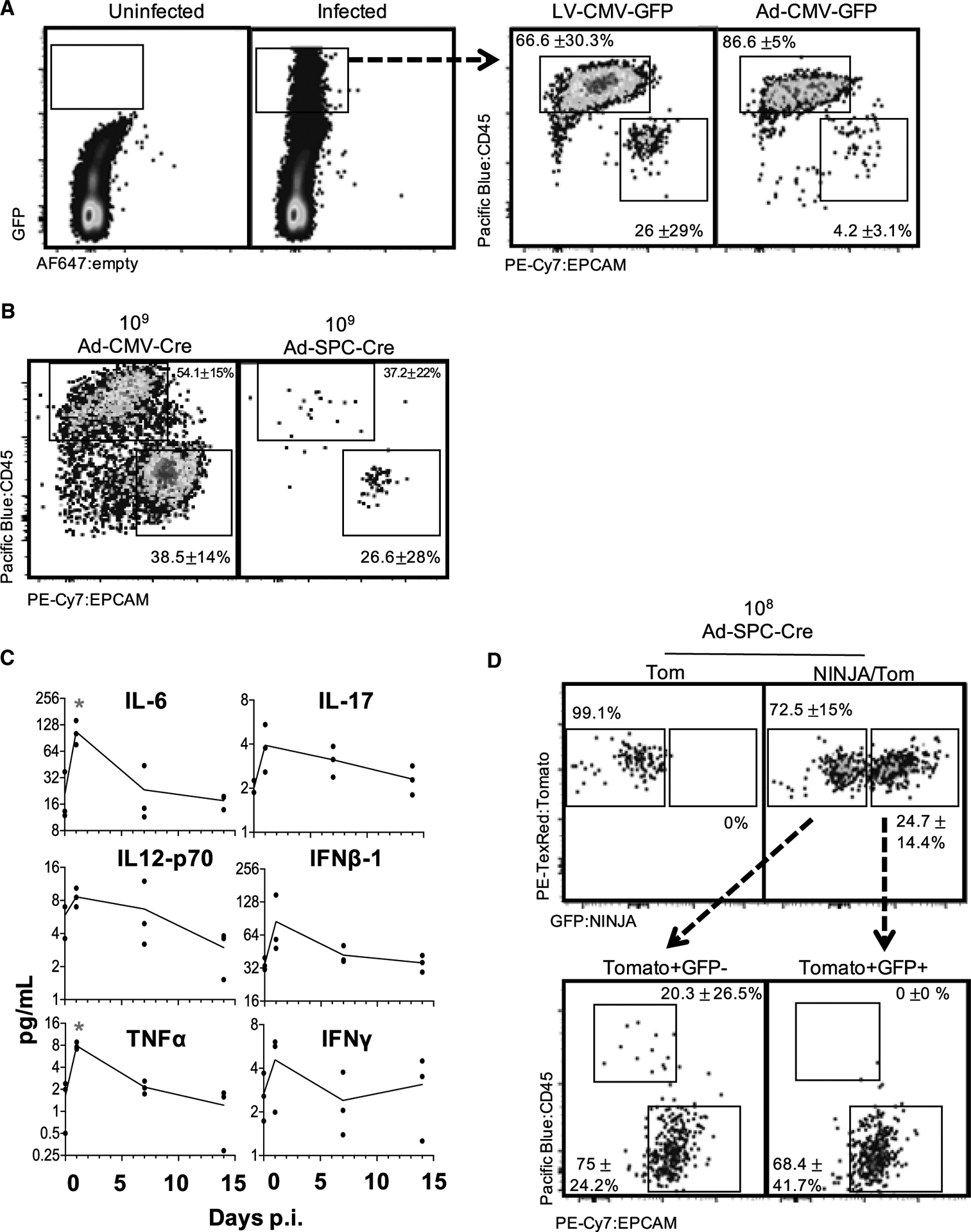
Neoantigen induction in KP-NINJA occurs in lung epithelial cells after overt inflammation is resolved (A) Infected (GFP+) cells 4–7 days after Lenti- or Adeno-CMV-GFP infection in lung, showing CD45+ and EPCAM+ populations. Percentages are average ± standard deviation; Lenti-CMV-GFP, n = 8 mice; Ad-CMV-GFP, n = 2 mice, from two independent repeats. (B) Infected (previously gated Tom+) lung cells gated for CD45 and EPCAM 4–7 days p.i. (high dose, 10^9^ PFU; percentages are average ± standard deviation; Ad-SPC-Cre, n = 4 mice; Ad-CMV-Cre, n = 5 mice, from two independent repeats). (C) Selected cytokines from Luminex cytokine panel, assessed for concentration in lung homogenate at 24 h, 7 days, or 14 days p.i. with 2.5 × 10^7^ PFU Adeno-SPC-Cre. n = 3 mice/time point. *p < 0.05, unpaired t test versus uninfected. (D) Expression of tomato and NINJA in T cell-depleted KP-NINJA/tomato (NINJA/Tom) lungs or KP-tomato control mouse (Tom) 21 days p.i., with mid-dose 10^8^ PFU Adeno-SPC-Cre and Dox/Tam administration. Percentages shown are out of all infected cells (Tom+) average ± standard deviation, n = 7. Arrows point to expression of EPCAM+ and CD45+ on cells from NINJA/Tom gates in the top right.

The KP × *NINJA* model uses infection with Cre-expressing viral vectors to initiate tumors and determine which cells can express the neoantigen (Figure 1A). Yet LV and adenoviral (Ad) vectors can infect many cell types in the lung (Shirley et al., 2020; Tippimanchai et al., 2018; Wilson et al., 2010). To assess the cell types infected by our i.t. administration method, we i.t. administered LV- and Ad-expressing GFP (LV-GFP [Lenti-CMV-GFP] and Ad-CMV-GFP, respectively). Four to 7 days later we analyzed the lung cells from infected mice by flow cytometry (fluorescence-activated cell sorting [FACS]) for the expression of GFP on EPCAM+ lung epithelial and CD45+ immune cells. Strikingly, ~66% of the LV-infected and ~87% of the Ad-infected cells were immune cells, with epithelial cells comprising the minority (26% and 4%, respectively) (Figure 2A). These findings are in line with previous studies (Tippimanchai et al., 2018; Wilson et al., 2010) showing LV and Ad vectors infect mostly alveolar macrophages and highlight a significant area of concern for neoantigen induction in the NINJA model, as the first step of regulation was dependent on which cell types expressed the Cre transgene.

The CMV promoter is universally expressed, so we next focused on limiting Cre activity using a tissue-specific promoter (the surfactant C promoter [SPC]) that would limit Cre expression to type II lung epithelial cells, which has the added benefit of increasing the fraction of KP LUADs (Sutherland et al., 2014; Tippimanchai et al., 2018). We compared the infection of *R26-LSL-Tomato* (Tom) mice with 2 × 10^8^–10^9^ plaque-forming units (PFU) of Ad-CMV-Cre or Ad-SPC-Cre and analyzed expression of CD45 and EPCAM on tomato+ cells in the lung. As with Ad-CMV-GFP, Ad-CMV-Cre showed infection of a majority CD45+ (54%) cells and 39% EPCAM cells, but by contrast, ~27% of the tomato+ cells in Ad-SPC-Cre-infected mice were EPCAM+, while only 37% were CD45+, with fewer cells expressing tomato overall (Figure 2B). These ratios of infected cells were much closer than we expected between the CMV-driven and SPC-driven Cre, and led to the realization that, while high doses (10^9^) of the vectors were used to identify the rare infected cell populations, epithelial cell recovery was impaired in those experiments, likely due to increased lung injury and fibrosis (Zhou et al., 2014). This damage was not observed in the 10^7^ PFU range and, in line with previous studies, we chose Ad-SPC-Cre at 10^7^ PFU for our tumor experiments (Ferone et al., 2016).

Administration of Ad vectors is associated with an inflammatory response (Shirley et al., 2020; Tippimanchai et al., 2018). To mitigate this, the NINJA model was designed to delay neoantigen induction until after the inflammation associated with Ad infection had subsided. To determine when inflammatory cytokines/chemokines abate following adenoviral infection, we infected mice with 2.5 × 10^7^ PFU Ad-SPC-Cre i.t. and isolated and processed lung tissue and serum at 0, 1, 7, and 14 days post infection (p.i.). Using tissue lysate, we performed Luminex analysis to assess the concentration of 44 cytokines and chemokines at each time point. Consistent with previous studies (Zhou et al., 2014), we found that nearly all inflammatory cytokines/chemokines assessed had returned to baseline by day 7 after adenoviral infection (Figure 2C; Table 1). The same mice had high variability in serum cytokine levels, but results were not significantly different at 7 days p.i. (Figure S1). Thus, we waited until day 7 p.i. to start the induction of neoantigens in KP-NINJA tumors.

### Testing neoantigen induction in KP-NINJA mice

To test the strategy of induction of NINJA, we bred KP × NINJA and KP × NINJA × Tom mice. NINJA was also designed to allow an extra layer of specificity for neoantigen induction by using tissue-specific promoters driving rtTA in tumor cell populations. For this, we crossed all our KP-NINJA containing mice to CCSP-rtTA (the “line 1” first-generation CCSP-rtTA is expressed in both type I and II epithelial cells, unlike later versions that are more specific for club cells; Perl et al., 2009). In KP × NINJA × Tom × CCSP-rtTA Tg mice (KP-NINJA/Tom), tumors are initiated by i.t. Ad-SPC-Cre, which activates Kras^G12D^ (K), eliminate p53 (P), and “poises” the FLPoER in the regulatory module of NINJA for subsequent activation by doxycycline and tamoxifen (Dox/Tam) treatment (Figure 1A). Dox treatment activates FLPoER expression in a CCSP-rtTA-dependent manner. Tam causes nuclear translocation of FLPoER and mediates a permanent inversion in the neoantigen module, leading to the expression of neoantigens derived from lymphocytic choriomeningitis virus (LCMV) glycoprotein (GP)-derived (GP33–43 and 66–77) from the universally expressed CAG promoter (Damo et al., 2020). Neoantigens are contained within a loop of GFP so that neoantigen expression can be read out by GFP fluorescence.

To determine the fraction of Cre-expressing GFP+ cells, we infected KP-NINJA/Tom mice and p53 × Tom (controls) with mid-dose 2 × 10^8^ PFU of Ad-SPC-Cre. After 7 days mice were treated with Dox (in food; days 7–10) and Tam (gavage; days 8–10), and we assessed the fraction of tomato+ GFP− and tomato+ GFP+ cells on day 21 after infection (which is on day 14 after NINJA neoantigen activation; Figure 2D). GFP was expressed by 25% of the tomato+ cells in the KP × NINJA × Tom mice, and none of the tomato+ GFP+ (neoantigen+) cells were CD45+. By contrast, 20% of the tomato+ GFP− cells were CD45+, in line with the fact that CCSP-rtTA helps restrict the expression of neoantigens to lung epithelial cells after Dox/Tam treatment in this model (Figure 2D). Thus, through tissue-specific promoters and tissue-restricted rtTA expression, the KP × NINJA × Tom model successfully limited neoantigen expression to lung epithelial cells and eliminated off-target sources of neoantigen that could drive confounding T cell responses. Note, these experiments had a large variability, and required a higher dose of Ad-SPC-Cre than that used for tumor induction. These higher doses were necessary to see the rare population of tomato-expressing cells, but could be the consequence of increased lung damage (Zhou et al., 2014).

### KP-NINJA mice form tumors with high penetrance and consistent neoantigen expression

**Figure 3.**
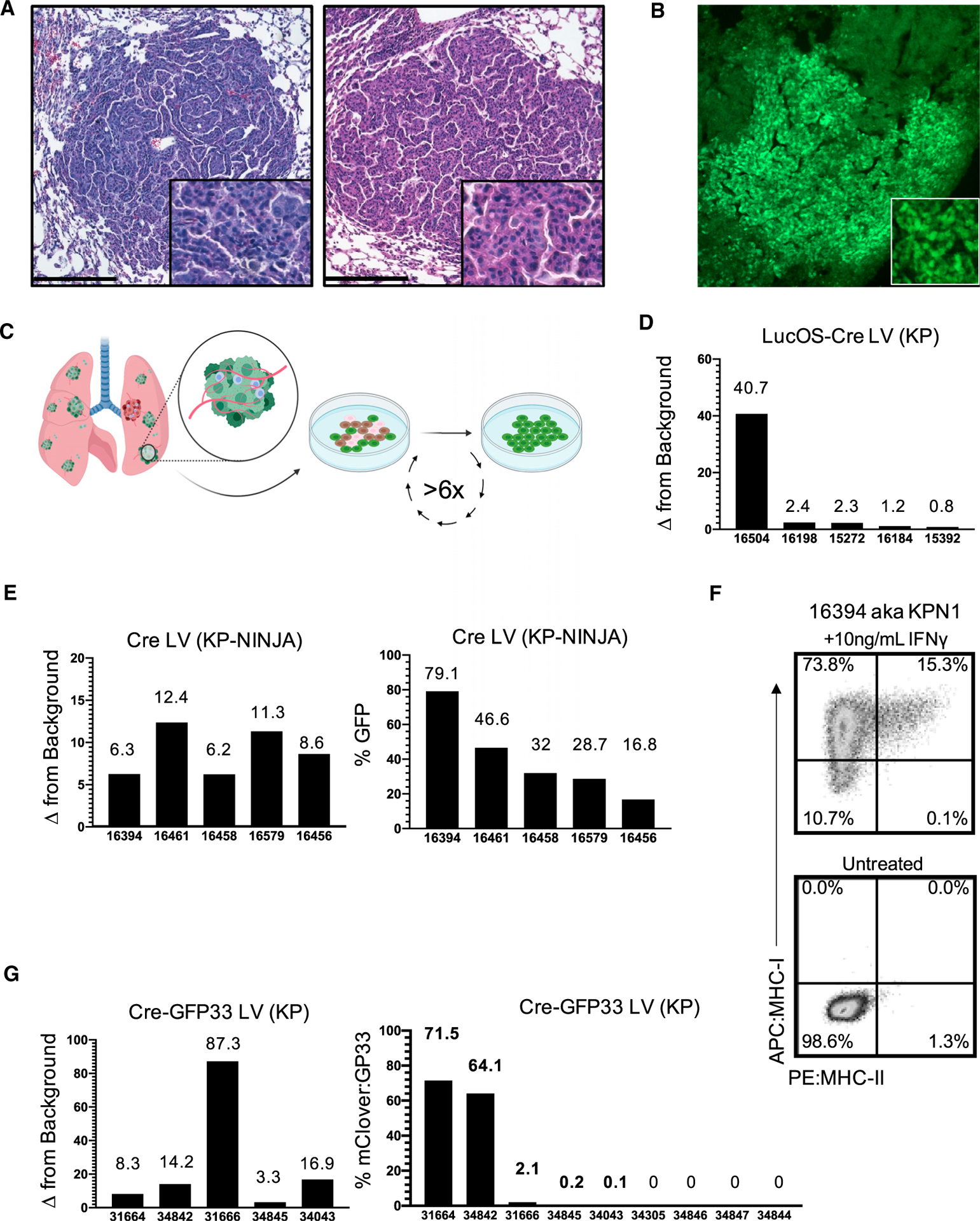
Antigen expression in KP-NINJA tumors has more uniform expression versus Lenti-neoantigen tumors (A) Representative H&E images of lung tumors 25 weeks p.i. Left panel: Adeno-SPC-Cre in KP-NINJA; representative of n = 15 tumors imaged (34/34 mice had tumors by necropsy, 100% penetrance). Right panel: LucOS-Cre LV (Lenti-ubc-LucOS-pgk-Cre) in KP; representative of n = 16 tumors imaged (20×). Scale bar, 200 μm. (B) Immunofluorescence image of KP-NINJA lung tumor. Green, endogenous NINJA-GFP (10×). (C) Schematic of cell line generation from primary lung tumors. All analysis occurred after a minimum of six passages in culture. (D) Fold change relative to background of neoantigen-luciferase intensity, in LucOS-Cre LV cell lines derived from 20 week p.i. KP lung tumors. (E) KP-NINJA cell lines. Left panel: fold change in NINJA-GFP expression is measured by geometric mean of GFP+/GFP− MFI. Right panel: percent of cells in each cell line positive for neoantigen expression by flow cytometry. Cre LV (Lenti-pgk-Cre) in KP-NINJA+ Dox/Tam lines from 8 week p.i. tumors. (F) MHC-I and MHC-II expression on a KP-NINJA cell line from (E) after overnight stimulation with IFNγ. A single representative experiment shown. (G) Cre-GFP33 LV cell lines. Left panel: fold change in GFP expression by MFI. Right panel: percentage of cells in each cell line positive for neoantigen expression by flow cytometry. Cre-GFP33 LV (Lenti-CMV-Cre-sv40-GP33) in KP lines from 8 week p.i. tumors.

To compare tumors and anti-tumor T cell responses between KP-NINJA and LV-programmed KP tumors, we infected KP × R26-NINJA/NINJA × CCSP-rtTA mice (henceforth called “KPNINJA” mice) with 2.5 × 10^7^ PFU Ad-SPC-Cre i.t. and then treated mice with Dox/Tam at days 7–10. As a comparison, we infected KP mice i.t. with LVs encoding luciferase-ovalbumin-SIY antigen and Cre (LucOS-Cre LV) (full construct with promoters: Lenti-UBC-LucOS-pgk-Cre) (DuPage et al., 2011). At 20–25 weeks p.i. (which is also 19–24 weeks after NINJA antigen induction; hereafter KP-NINJA mice will be referred to by the weeks following tumor induction, not antigen), we analyzed tumors from the lungs of mice by tissue histology (Figure 3A). LucOS-expressing KP tumors and KP-NINJA tumors had similar adenocarcinoma-like morphology. Moreover, all Ad-SPC-Cre-induced KP-NINJA mice had visible tumor lesions (34/34 mice), demonstrating that the KP-NINJA model develops tumors with 100% penetrance.

Next, we analyzed neoantigen expression by KP-NINJA tumors. By immunofluorescence (IF), most 20-week KP-NINJA tumors had GFP+ cells, demonstrating that these tumors maintained neoantigen expression throughout development (Figure 3B). However, the fraction of GFP+ and GFP− tumor cells was difficult to quantify by IF, particularly due to the low amount of GFP fluorescence from the NINJA construct (due to the inserted neoantigens) (Damo et al., 2020). A portion of N-terminal GFP is expressed in all tissues in the NINJA model, rendering commercially available anti-GFP antibodies ineffective. (Conformation-dependent antibodies can also be used to detect NINJA, but present difficulties with staining as they are currently mouse antibodies.) To quantify GFP expression by a more robust method, we generated independent lung tumor cell lines from individual KP-NINJA mice. For comparison, we also generated tumor cell lines from LucOS-Cre LV-infected KP mice. We generated lines from five 20-week KP-NINJA mice and five 20-week LucOS-Cre LV-induced KP mice and quantified neoantigen expression among the cell lines (Figure 3C). Luciferase assays showed a wide range of expression between LucOS-Cre LV-induced lines (~41×; Figure 3D). By contrast, the KP-NINJA lines exhibited an ~5× range of variance, with 16.8%–79.1% of the cells expressing GFP and 100% (5/5) of lines expressing antigen (Figure 3E). Moreover, because NINJA allowed for analysis of how much neoantigen was expressed by each tumor cell (mean fluorescence intensity [MFI] of GFP), we analyzed the GFP MFI among GFP+ tumor cells and all five cell lines fell within a 2-fold MFI range (Figure 3E). We also validated that all five KP-NINJA lines upregulated MHC class I after overnight stimulation with interferon gamma and thus maintained antigen presentation ability (Figures 3F and S2). This suggested that KP-NINJA tumors likely vary in the fraction of cells that are induced to express neoantigen (based on Dox/Tam exposure), but that, once NINJA was turned on, tumors expressed neoantigen with high consistency.

Because LucOS tumors did not allow us to measure the fraction of neoantigen+ tumor cells on a per cell basis like NINJA, it was difficult to know if the tumor cells within each line had differing LucOS expression (a mixture of strong and weak expressors) or if there was more uniform LucOS expression by all cells within a line. To separate these variables, we generated LVs that expressed Cre and a fluorescent neoantigen (the GFP variant mClover linked to the same LCMV GP33–80 antigen used in NINJA), which we refer to as Cre-GFP33 LV (full construct with promoters: Lenti-pgk-Cre-sv40-GFP33) (Figure S3) and used to generate tumors in KP mice. Analysis of cell lines from nine Cre-GFP33 LV-infected KP mice showed that only 5/9 (56%) lines had neoantigen-expressing cells, with an ~715-fold range of the percent of GFP+ cells between them (0.1%–71.5%; Figure 3G). Moreover, analysis of the MFI of GFP expression between the GFP+ cells in different lines also showed a range of expression (~26.5× range). Together, these data highlight the sources of variability in the LV-programmed antigenic KP tumor model and demonstrate how KP-NINJA reduces them.

### KP-NINJA tumors elicit anti-tumor CD8 T cell responses

**Figure 4.**
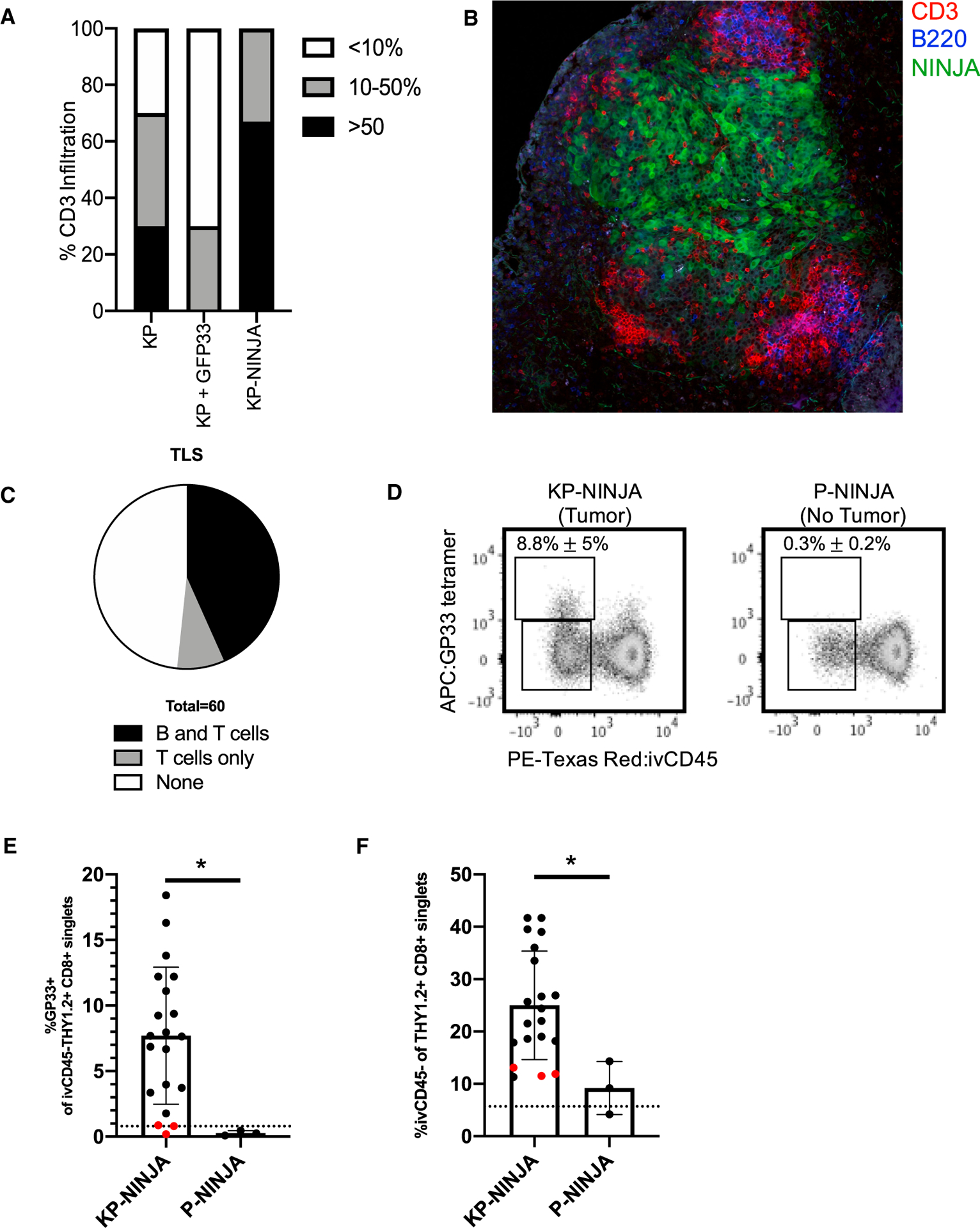
KP-NINJA tumors generate tumor neoantigen-specific T cell responses (A) Proportion of CD3 infiltration in tumors 8 weeks p.i. by IHC, blindly scored in KP Cre LV (KP) versus KP Cre-GFP33 LV (KP + GFP33) versus KP-NINJA Cre LV (KP-NINJA) for <10%, 10%–50%, or >50% tumor infiltration (n = 10 KP-NINJA, n = 12 KP + GFP33, n = 10 KP + Cre). (B) Immunofluorescence image of a KP-NINJA tumor. Green, endogenous NINJA-GFP; blue, B220 (B cells); red, CD3 (T cells) (40×, 4 × 4 tiled, 20 weeks p.i.). (C) Proportion of tumors scored in KP-NINJA that were associated with TLS; T cells only (5/60; 8.3%), T and B cells (26/60; 43.3%), or none (29/60; 48.3%). (D) Representative flow cytometry plots of Thy1.2+ CD8+ T cells gated for antigen specificity (GP33+) in tumor-bearing (KP-NINJA) or non-tumor-bearing (P-NINJA) lung. Note, i.v. CD45− indicates tissue infiltration. Percentages are averages ± standard deviation of n = 3 mice (P-NINJA) or n = 20 mice (KP-NINJA). (E) Quantification of lung-infiltrating antigen-specific T cells in (D) (%GP33+ i.v. CD45− Thy1.2+ CD8+) 8 weeks p.i. with Ad-SPC-Cre. The dotted line represents a control C57BL/6J mouse. Red dots represent mice where %GP33+ was not higher than the FMO control (3/20). *p < 0.05 in unpaired t test. (F) Quantification of total endogenous T cell infiltration in tumor-bearing or non-tumor-bearing lung in (D) (i.v. CD45− Th1.2+ CD8+). The dotted line represents a control C57BL/6J mouse. Red dots are the same mice as shown in (E). *p < 0.05 in unpaired t test.

To quantify whether KP-NINJA tumors were infiltrated by T cells, we performed anti-CD3 IHC staining on tumor-bearing lungs from 8-week KP-NINJA mice, and compared them with KP mice infected with Cre-GFP33 LV or non-antigenic Cre LV (full construct with promoters: Lenti-pgk-Cre). Despite the high penetrance of tumor formation at 20 weeks post Ad-SPC-Cre infection discussed above, it was difficult to identify tumors at the earlier 8-week time point by FFPE section. This was likely due to the relative difficulty of capturing the rarer tumor nodules in the Ad-SPC-Cre condition in 4-μm tissue sections (inducing tumors with Cre LV in both KP and KP-NINJA resulted in many tumors per lobe that were easily identifiable by H&E). We scored the available 8-week tumors blindly for infiltration based on the fraction of the tumor parenchyma that contained CD3+ cells (<10%, 10%–50%, or >50%). Consistent with their loss of antigens, 70% of Cre-GFP33 LV tumors (7/10) were poorly infiltrated by CD3+ cells (<10%) and none were heavily infiltrated (>50%; Figures 4A and S4). By contrast all KP-NINJA tumors had moderate to heavy infiltration by CD3+ cells, with 75% (8/12) falling into the latter category. We had also previously demonstrated that 20-week LucOS-Cre LV-induced tumors had tumor-associated tertiary lymphoid structures (TA-TLSs) marked by their association with B cell clusters (consisting of 20+ cells; Joshi et al., 2015). Analysis of 20-week KP-NINJA tumors by IF also showed the presence of peritumoral patches of CD3+ T cells and B220+ B cells, with 5/60 (8.3%) of tumors having clusters of just peritumoral T cells and 26/60 (43.3%) having T and B clusters (Figure 4C). These data are consistent with the idea that KP-NINJA tumors (like LucOS-Cre LV-induced tumors) could be associated with TA-TLSs, although in-depth analysis will be necessary to confirm this in the future.

Next, we assessed responses by tumor-specific T cells in KP-NINJA mice. We performed FACS analysis on 8-week p.i. mice to analyze lung tissues for the presence of GP33-specific CD8 T cells (identified by MHC class I tetramers). As lungs are highly vascularized, we utilized intravascular labeling with intravenously (i.v.) administered PE-Texas Red-labeled CD45 antibody, which marks cells in the circulation but not the lung parenchyma (Joshi et al., 2015). Analysis of P-NINJA mice (which lack the Kras allele and so cannot form tumors) revealed that mice did not have significantly increased infiltration of CD8+ T cells into their lung parenchyma (i.v. CD45−) or the presence of neoantigen-specific GP33-specific CD8+ T cells. By contrast, 20/20 KP-NINJA mice had increases in lung-infiltrating CD8+ T cells at 8 weeks p.i. (indicating 100% of tumors present), and ~9% of all CD8 T cells were lung parenchyma GP33-specific CD8 T cells (17/20 mice, 85%) (Figures 4D and 4E). These data demonstrate that KP-NINJA mice elicit tumor-dependent GP33-specific CD8 T cell responses with high penetrance.

### KP-NINJA tumor cell lines respond to immunotherapy

**Figure 5.**
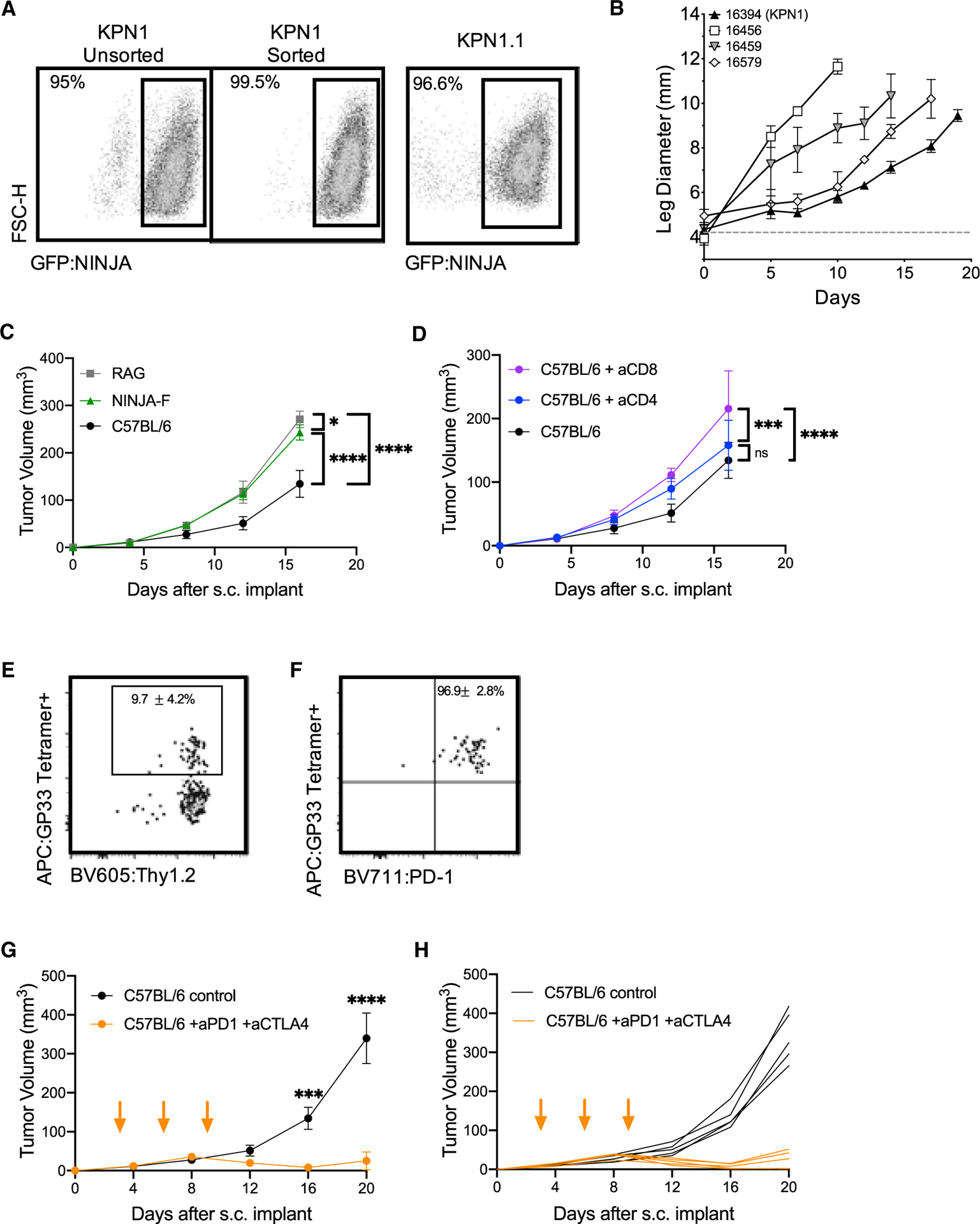
KP-NINJA tumor cell lines are immunogenic and respond to checkpoint therapy (A) Expression of NINJA-GFP in one of the KP-NINJA primary cell lines (16394, also known as KPN1). Left two panels: KPN1 before and after sorting for GFP+. Right panel: after one passage through C57BL/6J mice and rederivation of the cell line as KPN1.1. (B) Mean intramuscular growth of 200K cells of four transplanted primary KP-NINJA lung cell lines in NSG mice (16394, 16459, and 16579 all n = 3 mice; 16456 n = 5; error bars are SD). (C) Mean subcutaneous growth of 500K KPN1.1 in *Rag1*^−/−^, wild-type C57BL/6J, or NINJA-F (antigen-tolerant) mice. Representative of two independent experiments (RAG n = 5 mice, NINJA-F n = 8, C57BL/6J n = 10; the same C57BL/6J untreated control) is shown in (D). (D) Mean subcutaneous growth of 500K KPN1.1 in wild-type C57BL/6J with or without CD4 or CD8 depletion (200 μg/mouse/antibody were administered i.p. every 3 days, from day −3 until study endpoints, indicated on the x axis). aCD8 and aCD4 n = 5 mice each; C57BL/6J untreated control is also shown in (C). (E) Representative flow cytometry plots of CD8+ CD4− tumor-infiltrating T cells from KPN1.1 subcutaneous transplant tumors, gated for antigen specificity (GP33+). Percentages shown are averages ± standard deviation; n = 3 mice for tumors and n = 5 for lymph nodes (LNs). (F) PD1 expression on T cells from the GP33+ Thy1.2+ gate in (E). Percentages shown are averages ± standard deviation; n = 3 mice for tumors and n = 5 for LNs. (G) Mean subcutaneous growth of 500K KPN1.1 in wild-type C57BL/6J mice, with or without anti-PD1 200 μg and anti-CTLA4 200 μg dosing. Orange arrows indicate treatment; days 3, 6, and 9. Anti-PD1 + anti-CTLA4 n = 10 mice, C57BL/6J n = 10, from two independent experiments. (H) Individual tumor curves from (G). In (C), (D), (G), and (H), *p < 0.05, **p < 0.01, ***p < 0.001, ****p < 0.0001; two-way ANOVA.

Transplantable tumor models are useful for studies of new immunotherapy targets and testing combination therapies. We confirmed that our KP-NINJA cell lines were transformed by testing growth in NSG immunocompromised animals (Figure 5B). Next, we generated a cell line called “KPN1.1” by FACS sorting the 16394 cell line for GFP expression (Figure 5A) and passing tumors once through C57BL/6J mice subcutaneously (s.c.). Note, passage did not impact GFP expression or lead to loss of MHC expression (data not shown), but did yield tumors that grew consistently after s.c. transplant. To assess the impact of tumor-specific T cells on the growth of KPN1.1 tumors, we transplanted KPN1.1 tumors into C57BL/6J, *Rag1*^−/−^, “4get” GFP-tolerant mice, or NINJA(F) mice, which express NINJA in all tissues and are tolerant to GFP and the neoantigens) (Damo et al., 2020). KPN1.1 tumors grew significantly faster in *Rag1*^−/−^ and NINJA(F) mice than in C57BL/6J mice, but not in GFP-tolerant mice, consistent with immune-mediated tumor control that is dependent on the neoantigens in NINJA (Figures 5C, S5A, and S5B; and in an antigen-negative control KP lung line, Figure S5C). We also observed that the immune-mediated delay in KPN1.1 tumor growth was dependent upon CD8 T cells, but not CD4 or B cells (Figures 5D and S5D; data not shown). Analysis of tumor-infiltrating GP33-specific CD8 T cells showed high PD-1 expression (~97% of GP33+ cells) (Figures 5E and 5F). Thus, we next asked whether immune responses could control the growth of KPN1.1 tumors when mice were treated with anti-PD1 and anti-CTLA4 combination therapy (combo CPI). Combo CPI and anti-CTLA4 alone had the strongest impact on tumor growth, with anti-PD1 alone having a less dramatic but still significant impact on growth. There were higher numbers of complete regressions in combo CPI (6/9 mice) compared with single therapy (1/10 anti-PD1, 1/10 anti-CTLA4) (Figures 5G, 5H, and S5E–S5I). Together these data clearly demonstrate that neoantigen programming with NINJA yields immunogenic tumors that are responsive to checkpoint blockade therapy.

## DISCUSSION

We have demonstrated that the novel KP-NINJA mouse is an autochthonous, immunogenic LUAD model that improves upon previous versions of the KP model for studies of cancer immunology. We have also generated the KPN1.1 tumor cell line and demonstrated that it is responsive to aCTLA4 and aPD1 therapy. We believe that both of these models will be broadly useful for investigators studying immunotherapeutic responses against Kras-driven LUAD.

Lung cancer remains lethal, with a 5-year survival rate of only 21% (Siegel et al., 2021). Research in LUAD is critical to increase our understanding of the underlying biology of the disease and responses to therapy, and the most powerful current approach requires relevant animal models of disease. Genetically engineered autochthonous mouse models of lung cancer have the advantage of defined alterations that are relevant to patient disease, a timeline of natural development toward metastasis that mimics patient progression, and the ability to model many of the relevant features of human disease, including natural tumor microenvironments and architecture (Kellar et al., 2015; Rehm et al., 1991). However, the adaptive immune responses against lung tumors in traditional GEMMs are poor, as their low mutation burden results in a very low baseline of anti-tumor immune response (McFadden et al., 2016). They are generally not responsive to checkpoint therapy (Pfirschke et al., 2016). Efforts to increase the immunogenicity of the autochthonous lung cancer models have relied on both carcinogens and neoantigens. Both play a role in lung cancer research, but fill different niches. Neoantigen models excel at answering questions that require comparing defined responses between mice and between experiments. Carcinogen models better reflect the heterogeneity of disease in patients. Critically, both types of models are needed, and the choice of model will depend on the research question being addressed. Neoantigen models will be particularly useful for investigators who are interested in comparing tumor-specific CD8 T cells in the context of defined manipulations, such as therapies, or genetic manipulations in the host or tumor cells, etc.

When developing KP-NINJA, we evaluated three common approaches to neoantigen programming. The first is through the use of genomically encoded inducible neoantigens (Cheung et al., 2008; Schietinger et al., 2016). A challenge with these approaches has been the development of central tolerance toward the “neoantigen” prior to induced expression in the tumor, which necessitates the use of TCR transgenic T cells. While TCR transgenic T cells are powerful tools, they are often representative only of the biology of high-affinity T cells. In contrast, KP-NINJA allows for studies with endogenous cells that may encompass a broad range of antigen sensitivity. A second method is intratumoral electroporation of neoantigen cDNA after regulatory T cell depletion (Radkevich-Brown et al., 2010). This therapeutic *in situ* induction of antigen inhibited growth of preexisting tumors and protected against tumor cell rechallenge. However, due to the necessity of preexisting tumors, this model is more appropriate for questions of therapy and cancer vaccines, rather than the study of early tumor-T cell interactions. A third neoantigen-programming method uses lentiviral- or transposon-based delivery of neoantigens along with the tumor-initiating recombinases or oncogenes. This method succeeds in generating immunogenic tumors and T cell responses, but there are limits to the types of questions that can be investigated in these systems. Early “tumor-specific” T cell responses may be primed by LV-infected myeloid cells rather than tumor cells (DuPage et al., 2011). The amount of neoantigen expressed in each tumor cell varies greatly, and LV proviruses are susceptible to silencing after incorporation into the genome (this depends greatly on the promoters; CMV, sv40, and other viral promoters are prone to silencing compared with non-viral UBC and PGK). Promoter silencing is a likely explanation for the absence of antigen expression in our model, as the GFP33 is driven by the sv40 promoter (Hino et al., 2004; Muller-Kuller et al., 2015; Pannell et al., 2000; Pfeifer et al., 2001; Yao et al., 2004). Lentiviral vectors also have a broad tropism, and infected “bystander” CD45+ cells could express neoantigens and persist up to 1 year post transduction (Wilson et al., 2010). Finally, LVs are limited by the ease of *in vivo* delivery, requiring surgery to initiate tumors at sites relevant for non-lung tumors (i.e., pancreas).

Compared with the above methods, KP-NINJA provides a unique and compelling tool to fill this gap and study anti-tumor responses in an autochthonous tumor model and matched cell lines for transplant studies. The KP-NINJA mouse maintains the disease relevance of the KP lung cancer model (Jackson et al., 2005) while forming immunogenic lung tumors that generate robust endogenous neoantigen-specific T cell responses. The unique regulation of the NINJA system prior to induction avoids the central or peripheral tolerance observed in other inducible antigen models (Damo et al., 2020), while the temporal uncoupling of tumor induction (via Cre recombinase) from antigen expression (via systemic Dox/Tam administration) allows for the clearance of overt inflammation prior to antigen expression (or even neoantigen induction at later stages of disease), and meanwhile ensures that neoantigen-specific T cell responses require the development of tumors. This creates the potential for early tumor-specific T cell analysis without the obvious caveats presented by other models. The germline NINJA neoantigen also has the benefit of consistent and predictable neoantigen expression from each induced tumor cell, as it is not subject to the variations of expression seen in LVs due to genomic location or copy number. This consistent level of antigen expression is critical to be able to compare T cell responses between experiments and particularly to ask questions regarding T cell rates of differentiation in different tumor environments and under checkpoint therapies, as differing levels of antigen availability can have dramatic impacts on T cell fate (Kim and Williams, 2010). Variability in neoantigen expression, in both the primary tumor and in cell lines, could present possible complications for reproducible investigations of immune interactions in tumor models, but the KP-NINJA model minimizes this. Finally, our use of Ad-SPC-Cre and CCSP-rtTA Tg led to expression that was restricted to EPCAM+ lung epithelial cells, which contrasted to the promiscuous expression in LV-programmed models.

The consistent expression of a neoantigen between tumors in KP-NINJA is ideal for studies of cancer immunoediting. Moreover, because the antigenic epitopes are contained in the GFP protein itself, MFI of GFP can be used to quantify the amount of neoantigen that each tumor cell expresses by FACS. KP-NINJA/Tom mice allow for analysis of the % of GFP+ among tomato+ cells at early time points, easily distinguishing the infected and neoantigen-expressing cells. They confer the capacity to investigate the impact, speed, and mechanism of immunoediting in future studies of LUAD. Mixed tumor antigenicity (i.e., 50:50 GFP+:GFP−) may be desired for applications such as comparisons of gene expression in antigenic or non-antigenic tumors (i.e., via laser capture microdissection or protein expression by IHC). This mixed immunogenicity model may also more accurately reflect physiology and outcomes in the diverse mutation landscape of human adenocarcinomas, where not all patient tumors contain identical mutational burden. Dox/Tam dosing could be altered to potentially increase or decrease the fraction of neoantigen+ tumor cells, or if 100% antigen expression is desired, FLPo-inducible Kras^FSF–G12D^p53^frt/frt^ (Lee et al., 2012) mice could be bred to NINJA × Cag-rtTA Tg to generate KP^FRT^-NINJA mice, so that neoantigen expression and tumor transformation could be initiated simultaneously by Dox/Tam induction, but remain temporally delayed from the inflammatory i.t. infection with Cre.

Finally, NINJA mice have the potential to be used in all GEMM models with (1) Cre-inducible promoters in different organs (i.e., KP × PDX1-CreER for pancreas), and (2) lox-stop-lox “floxed” mutations (i.e., Braf CA-V600E Pten^flox/flox^ for melanoma). This would allow for the study of tumor antigen-specific T cells responses in other types of cancer. In line with this, our preliminary data suggest that NINJA-expressing tumors are also immunogenic when derived from PDAC (KP), soft-tissue sarcoma (KP), and melanoma (Braf CA-V600E Pten ^flox/flox^). Thus, the NINJA platform provides great flexibility for researchers interested in studies of cancer immunology in lung and potentially other cancer types.

### Limitations of the study

- KP-NINJA tumors were induced with a consistent Dox/Tam dose, but the dose was not optimized. Thus, we did not determine if it was possible to generate 100% neoantigen+ tumors.
- The KPN1.1 cell line was derived from a Cre LV-infected Dox/Tam-treated KP-NINJA mouse. Thus, there is constitutive Cre expression in these lines, which some studies suggest can be toxic (Loonstra et al., 2001). Ad-SPC-Cre-induced primary cell lines would not maintain Cre expression.
- We did not characterize the components of TLS beyond the presence of clusters of B220+ and CD3+ cells. LucOS-Cre LV-induced TLS contain many cell types (i.e., follicular dendritic cells, fibroblastic reticular cells, and high endothelial venules, etc.) and markers (CXCL13, CCL21/19, etc.) that define the TLS and further work will be required to assess if these elements are also present in KP-NINJA TLS.
- The NINJA-inducible antigen system contains a limited number of known antigens. To mimic the tumor mutational burden in human disease, other models, such as mutagen exposure or deficiency in mismatch repair genes, may be preferred.

## STAR★METHODS

### RESOURCE AVAILABILITY

#### Lead contact

Further information and requests for resources and reagents should be directed to and will be fulfilled by the lead contact, Nikhil Joshi (nikhil.joshi@yale.edu)

#### Materials availability

Mice strains generated in this study will be deposited to Jackson Laboratory.

#### Data and code availability

- All data reported in this paper will be shared by the lead contact upon request.
- This paper does not report original code.

### EXPERIMENTAL MODEL AND SUBJECT DETAILS

All studies were carried out in accordance with procedures approved by the Institutional Animal Care and Use Committee of Yale University. All mice were bred in specific pathogen-free conditions. For experiments, 6+ week old mice were used. Both male and female mice were used for experiments. C57BL/6J mice (Jackson Laboratories), NINJA-F mice (Damo et al., 2020), RAG mice (Jackson Laboratories) and Il4GFP [4get] mice (Weinstein et al., 2016) were used for all transplant experiments. KP × CCSP-rtTA mice, referred to here as KP mice, were obtained from Tyler Jacks and crossed to NINJA mice to obtain KP-NINJA (Kras^LSL-G12D/+^, p53^fl/fl^, R26-NINJA/NINJA, CCSP-rtTA^+^) mice. R26-LSL-Tomato mice (Jackson Laboratories) were crossed to KP or KP-NINJA mice to obtain KP-Tom or KP-NINJA/Tom.

Growth conditions for all primary lung cell lines generated in this study are 37C and 5% CO2 in DMEM + 10% FBS + 1% Pen/Strep.

**Table:** 

Experimental Models: Cell Lines		
16394 (KPN1)	Cre LV + KP-NINJA mouse	Female
KPN1.1	16394 (KPN1) after subcutaneous tumor transplant (F1)	Female
16456	Cre LV + KP-NINJA mouse	Female
16458	Cre LV + KP-NINJA mouse	Female
16461	Cre LV + KP-NINJA mouse	Female
16504	LucOS Cre LV + KP mouse	Female
16198	LucOS Cre LV + KP mouse	Female
15272	LucOS Cre LV + KP mouse	Female
16184	LucOS Cre LV + KP mouse	Female
15392	LucOS Cre LV + KP mouse	Female
31664	Cre GFP33 LV + KP mouse	Female
34842	Cre GFP33 LV + KP mouse	Female
31666	Cre GFP33 LV + KP mouse	Female
34845	Cre GFP33 LV + KP mouse	Female
34043	Cre GFP33 LV + KP mouse	Female

### METHOD DETAILS

#### Lentiviral vector production and administration

“GFP33-Cre LV” plasmids were generated by cloning GFP33 into Cre LV. Lentiviral vectors were produced by transfecting 293FS* cells with GFP33-Cre LV, PSPax2 and VSV-G vectors at a 4:3:1 ratio, of which supernatant was harvested at 100,000g for 2 hours (20°C), after 32-hour incubation at 37°C. LV titer was quantified by transducing GreenGo cell line (10^5^ cells/well) in serial dilutions to 1mL of final volume and calculated with the formula: (% fluorescent cells × 10^5^ × Dilution Factor)/100 (pfu/mL).

#### Intratracheal infections

For autocthonous tumor generation KP-NINJA mice were infected intratracheally with 2.5 × 10^7−9^ PFU Ad5mSPC-Cre (Dr. Anton Berns, Netherlands Cancer Institute (a.berns2@nki.nl)), after precipitation with 10mM CaCl_2_ for 20–60 minutes, or 2.5 × 10^4^ PFU Lenti-cre or GFP33-Cre LV. To induce expression of NINJA neoantigen in infected cells, mice were given doxycycline hyclate chow (625mg/kg; Envigo cat. TD.09628) days 7–10 post infection (p.i.) and concomitantly treated with 4.4mg tamoxifen (MP Biomedicals cat. MP215673894) in corn oil (ThermoFisher Scientific cat. S25271) by gavage on days 8–10 p.i.

For lung expression quantification C57BL/6J mice were infected intratracheally with 2.5 × 10^7^ PFU GFP LV. KP-Tom mice were infected intratracheally with 2.5 × 10^8−9^ PFU Ad5mSPC-Cre (Dr. Anton Berns, Netherlands Cancer Institute (a.berns2@nki.nl)), after precipitation with 10mM CaCl_2_ for 20–60 minutes. Note, detecting Ad-SPC-Cre infected cells required higher doses of virus, but this dose of adenovirus has been associated with significant lung pathology (Zhou et al., 2014). Thus, for tumor initiation, we used lower doses as previously (Sutherland et al., 2014).

#### Lung and LN processing for flow cytometry

Lung was harvested from all mice, some used for IHC or IF, and the remaining lobes were processed as previously described (Joshi et al., 2007). Mediastinal lymph nodes were concomitantly harvested from all tumor-bearing mice. Cells were counted using a hemocytometer or using BD Cytoflex. Single cell suspensions were stained using one of the listed antibody panels in addition to tetramer for H2Db/GP33–43-specific CD8 T cells (NIH Tetramer Core Facility). Cells were stained using antibodies listed in Table S1 in FACs Buffer (2% FBS, PBS 1X without Mg^2+^/Ca^2+^). For intracellular staining, FoxP3/Transcription Factor Staining Buffer set (eBioscience cat# 00-5523-00) was used as per manufacturer’s protocol. Otherwise, cells were fixed in 1–2% paraformaldehyde (PFA) in 1X PBS at 4°C for 15 minutes. Cells were washed and resuspended in FACs Buffer until analysis on a BD LSRII flow cytometer (BD Biosciences).

#### Luminex assay/cytokine array

C57BL/6J mice were infected intratracheally with 2.5 × 10^7^ PFU Ad5mSPC-Cre (Dr. Anton Berns, Netherlands Cancer Institute (a.berns2@nki.nl)), after precipitation with 10mM CaCl_2_ for 20–60 minutes, and harvested on day 0, 1, 7, and 14 p.i.

Serum Isolation: Blood was collected retro orbitally, coagulated at room temperature for >30 minutes, and centrifuged at 1000xg for 10 minutes. Serum was diluted with sterile 1X phosphate buffered saline (PBS; ThermoFisher scientific cat. 10010023) per manufacturer instructions and frozen at −80 C. Lung homogenates: Whole lung tissue was removed following perfusion with 1X PBS, mechanically dissociated with scissors and homogenized with MP FastPrep 24–5g machine (MP Biomedicals cat. MP116913500) in 700 μL of RIPA 1x + HALT protease and phosphatase inhibitor (ThermoFisher Scientific cat. 89901, cat. 78440) on default mouse lung protocol. Samples were centrifuged at 1000xg for 10 minutes and protein was adjusted to 4 mg/mL after BCA protein assay (ThermoFisher Scientific cat. 23227). Samples were stored at −80 C until analysis. Serum and lung homogenates were analyzed by Eve Technologies Corporation using their “Mouse Cytokine Array/Chemokine Array” 44-Plex assay.

#### In vitro MHC-I/PDL1 expression

When cell lines are 70% confluent, add 10–20ng/mL Recombinant Murine IFN-gamma (Peprotech Cat# 315–05) in media. Incubate 16–18 hours and briefly wash with cold PBS 1x, add cold Trypsin for 30 seconds, gently pipette to dissociate, then proceed with flow cytometry staining as described previously.

#### Histology

Tissues were fixed in paraformaldehyde-lysine-periodate fixative (PLP), or 1x Formalin solutions in PBS (Millipore-Sigma) for 24–96 hours at 4°C, switched into 70% ETOH, and submitted to Yale histology core for paraffin embedding, hematoxylin and eosin (H&E) staining, and sectioning. H&E stained sections were imaged on a Nikon TE2000 microscope (Micro Video Instruments, Avon, MA) or on an EVOS FL Auto 2D microscope using a 20x objective. For anti-CD3 staining, unstained paraffin sections were stained as described previously (Connolly et al., 2021).

#### Immunofluorescence

Lungs were inflated with and fixed in paraformaldehyde-lysine-periodate fixative (PLP) overnight, placed in 30% sucrose solution in water for 4–8 hours, inflated with 30% Tissue Plus Optimal Cutting Temperature (OCT) compound (Fisher Scientific Cat. 23-730-571) in PBS, and frozen in OCT media at −80C.

Tissue sections were cut 20 μm thick in a cryostat (Leica CM 1850UV) at −20°C and stained with an anti-mouse CD3 (clone 17A2)-eFluor 660 antibody (ThermoFisher Scientific, cat: 50-0032-82) and Antifade Mounting Medium with DAPI (Vector Laboratories, cat: H-1200). Images were acquired using the EVOS FL AUTO 2.0 Imaging System at 10x magnification. Channels were overlayed using FIJI software (Schindelin et al., 2012).

#### Luciferase expression assay

500,000 cells from each cell line were plated in a 96 well plate, at least three wells apart to reduce interference. Cells were lysed using the Promega Luciferase Assay System (Promega Cat. E1500) according to manufacturer’s instructions. Background luciferase expression was measured on a luminometer prior to addition of the luciferase substrate, and then luciferase activity was measured. Cells were normalized to background and luciferase negative control line.

#### Primary tumor cell lines

Lungs of all mice were harvested 20 weeks post infection, digested in sterile MACs Dissociator C-tubes with 3 ml digestion media (0.03% Trypsin-EDTA Millipore-Sigma Cat. SM-2003-C, 3.125mg/ml Collagenase IV Worthington Biochem Cat. LS004189 in HBSS free media) for 40 minutes at 37C in rotating incubator. Homogenate was filtered through a cell strainer (Corning cat. 352340) and centrifuged at 200xg for 4 minutes at room temperature. Pellet was resuspended and cultured in 35mm plate at 37°C and 5% CO2 in complete DMEM (DMEM + 10% FBS + 1% P/S), + 1x Gentamicin (Gibco Cat. 15710064).

The KPN1 cell line was later sorted (SH800S, Sony Biotechnologies) to obtain a 100% GFP positive line before being subcutaneously injected in C57BL/6J mice. Tumors were then allowed to grow out to ~700mm^3 prior to being sterilely harvested as described above, to derive the KPN1.1 line. The non-antigenic KP control cell line “LGKP” was a gift from Tyler Jacks.

#### Tumor cell line transplants

Established cells were maintained in complete DMEM (10% HI-FBS, 55μM beta-mercaptoethanol, 1x Pen/Strep and 1x L-Glut). Prior to injection, cells were washed 3x with 1xPBS.

200,000 cells for each line were injected subcutaneously into the flank of NSG mice and tumor size was measured throughout the experimental time with a digital caliper (Thomas Scientific, catalog no. 1235C55) on two axes. Volume was calculated as: (X diameter in mm) × (Y diameter in mm) × (average of both measurements) = volume in mm^3^.

500,000 KPN1.1 cells were injected subcutaneously into the flank of NINJA(F), *Rag1*^−/−^, or C57BL/6J mice and tumor size was measured as described above. For T cell depletion, anti-CD4 (Clone: GK1.5) 200ug or anti-CD8 (Clone: 53–6.7) 200ug were injected intraperitoneally on day −3 and subsequently every 3 days until study end points. For checkpoint therapy, anti-PD1 200ug + anti-CTLA4 200ug were injected intraperitoneally on days 3, 6, and 9.

### QUANTIFICATION AND STATISTICAL ANALYSIS

GraphPad Prism and Microsoft Excel were used for statistical analysis. Details for each experiment are found in the figure legends.

### ADDITIONAL RESOURCES

Protocols for NINJA genotyping are available at http://www.nikjoshilab.org/protocols.

## Supplementary Material

1

2

## Figures and Tables

**Table 1.** Changes in lung cytokine expression following infection

Cytokine	24 h	7 days	14 days
IL-5	9.3	1.0	1.0
G-CSF	6.2	1.0	1.8
IP-10	6.2	1.5	0.8
IL-6	5.1*	1.1	0.8
TNF-a	4.8*	1.3	0.7
MIP-1a	3.2*	1.4	1.1
TimpI	2.9*	1.3	1.1
IL-4	2.8	1.2	0.8
IFNB-1	2.4	1.2	1.0
LIF	2.2*	1.2	1.2
MIP-3a	2.1*	1.5*	1.4
IL12 p40	2.0*	2.5*	1.9
MIG	1.9	1.6	0.9
MIP-1b	1.8	1.2	1.4
IL-1b	1.8*	0.9	0.7*
IFNg	1.7	0.9	1.2
MIP-2	1.7	0.9	0.8
MCP-5	1.6*	1.6*	1.3
TARC	1.6	1.4	1.3
IL-1a	1.5	0.9	0.7
IL12 p70	1.5	1.1	0.5
GM-CSF	1.4*	0.9	0.6*
MDC	1.4	1.3	1.1
M-CSF	1.3	1.0	0.8
IL-17	1.1	1.4	1.1

Relative fold change from baseline of lung tissue cytokine levels 24 h, 7 days, and 14 days after infection with 2.5 × 10^7^ PFU Ad-SPC-Cre.

* p < 0.05 unpaired t test versus uninfected. n = 3 mice/time point.

**Table:** KEY RESOURCES TABLE

REAGENT or RESOURCE	SOURCE	IDENTIFIER
Antibodies		
EpCam/CD326-PeCy7 (clone: G8.8)	BioLegend	Cat# 118216; RRID:AB_1236471
CD45-Pacific Blue (clone:30-F11)	BioLegend	Cat# 103125; RRID:AB_493536
CD31-BV605 (clone:390)	BioLegend	Cat# 104247; RRID:AB_2563982
CD45-PECF594 (clone:30-F11)	BD Biosciences	Cat# 562420; RRID:AB_11154401
CD8-PECy5 (clone:CT-CD8a)	Thermo Fisher Scientific	Cat# MA5-17601; RRID:AB_2538991
CX3CR1-BV711 (clone: SA011F11)	Biolegend	Cat# 149031; RRID:AB_2565939
Thy1.2-BV605 (clone:30-H12)	BioLegend	Cat# 105343; RRID:AB_2632889
Tim3-PECy7 (clone:RMT3-23)	BioLegend	Cat# 119716; RRID:AB_2571933
PD-1-BV421 (clone:29F.1A12)	BioLegend	Cat# 135217; RRID:AB_10900085
SLAMF6-PE (clone:330-AJ)	BioLegend	Cat# 134606; RRID:AB_2188095
TCF1-AF488 (clone: 812145)	R&D Systems	Cat# FAB8224R; RRID:AB_2888931
CD44-BV711 (clone:IM7)	BD Biosciences	Cat# 563971; RRID:AB_2738518
CD4-PERCP (RM4-5)	BioLegend	Cat# 100537; RRID:AB_893331
CD8a-BUV395 (53-6.7)	BD Biosciences	Cat# 565968; RRID:AB_2739421
PD1-BV711 (EH12.2H7)	BioLegend	Cat# 564017; RRID:AB_2738543
MHC Class I-APC (clone: H2-kB)	eBioscience	Cat# 17-5958-82; RRID:AB_1311280
PDL1-PE (clone:10F.9G2)	BioLegend	Cat# 124308; RRID:AB_2073556
CD62L-BV421 (clone: MEL-14)	BD Biosciences	Cat# 562910; RRID:AB_2737885
SlamF6-BV750 (clone: 13G3)	BD Biosciences	Cat# 747169; RRID:AB_2871904
Granzyme B-PE (clone: GB11)	BD Biosciences	Cat# 561142; RRID:AB_10561690
CD127-PE Dazzle (clone: A7R34)	BioLegend	Cat# 12-1271-82; RRID:AB_465844
TCF1-AF488 (clone: C63D9)	Cell Signaling Technologies	Cat# 6444; RRID:AB_2797627
Bacterial and virus strains		
Ad5CMVCre	UI Viral Vector Core	VVC-U of Iowa-5
Ad5mSPC-Cre	UI Viral Vector Core	VVC-Berns-1168
Ad5CMVhr-GFP	UI Viral Vector Core	VVC-U of Iowa-2161
Cre-GFP33 LV	This article	N/A
Cre LV	DuPage et al. (2011)	N/A
Critical commercial assays		
Mouse Cytokine Array/Chemokine Array: 44-Plex assay	Eve Technologies Corporation	MD44
Experimental models: Cell lines		
16394 (KPN1)	This article	N/A
KPN1.1	This article	N/A
16456	This article	N/A
16458	This article	N/A
16461	This article	N/A
16504	This article	N/A
16198	This article	N/A
15272	This article	N/A
16184	This article	N/A
15392	This article	N/A
31664	This article	N/A
34842	This article	N/A
31666	This article	N/A
34845	This article	N/A
34043	This article	N/A
Experimental models: Organisms/strains		
Mouse: C57BL/6J	The Jackson Laboratory	RRID:IMSR_JAX:000664
Mouse: Rag1−/−; B6.129S7-Rag1/J	The Jackson Laboratory	RRID:IMSR_JAX:002096
Mouse: KP-NINJA	This article	N/A
Mouse: KP-NINJA/Ai14	This article	N/A
Mouse: NINJA-F	Damo et al., 2020	N/A
Mouse: B6.129-*Kras*^*tm4Tyj*^*Trp53*^*tm1Brn*^/J	The Jackson Laboratory	RRID:IMSR_JAX:032435
Mouse: Il4GFP [4get]	Weinstein et al. (2016)	N/A

## Highlights

- A genetically encoded mouse model of tumor-immune interaction in Kras-driven LUAD
- Allows temporal and spatial control of tumor neoantigen expression
- Quantifiable and specific anti-tumor CD8 T cell responses are generated
- Tumor transplants from this model are responsive to combined aCTLA4+ aPD1 therapy

## MOTIVATION

The Kras-Lox-STOP-Lox-G12D p53 flox/flox (KP) mouse model faithfully recapitulates human LUAD but does not elicit tumor-specific T cell responses because developing tumors lack neoantigens. *In vivo* lentiviral transduction has been used to express neoantigens in KP LUADs, but this strategy has limitations, including infection-associated inflammation, neoantigen expression in non-tumor cells, and neoantigen silencing. The model described in this article provides an immunogenic alternative for the study of immune responses and checkpoint therapy in LUAD.
