# Supplementary material for: A mouse model for the study of anti-tumor T cell responses in Kras-driven lung adenocarcinoma
Source: Cell Rep Methods. 2021 Sep 16;1(5):100080. doi: 10.1016/j.crmeth.2021.100080 (PMC8500377; doi:10.1016/j.crmeth.2021.100080)
Supplement: Document S1. Figures S1–S5 and Table S1 [file mmc1.pdf]

Cell Reports Methods, Volume *1*

Supplemental information

A mouse model for the study of anti-tumor T cell responses in Kras-driven lung adenocarcinoma

Brittany Fitzgerald, Kelli A. Connolly, Can Cui, Eric Fagerberg, Dylan L. Mariuzza, Noah I. Hornick, Gena G. Foster, Ivana William, Julie F. Cheung, and Nikhil S. Joshi

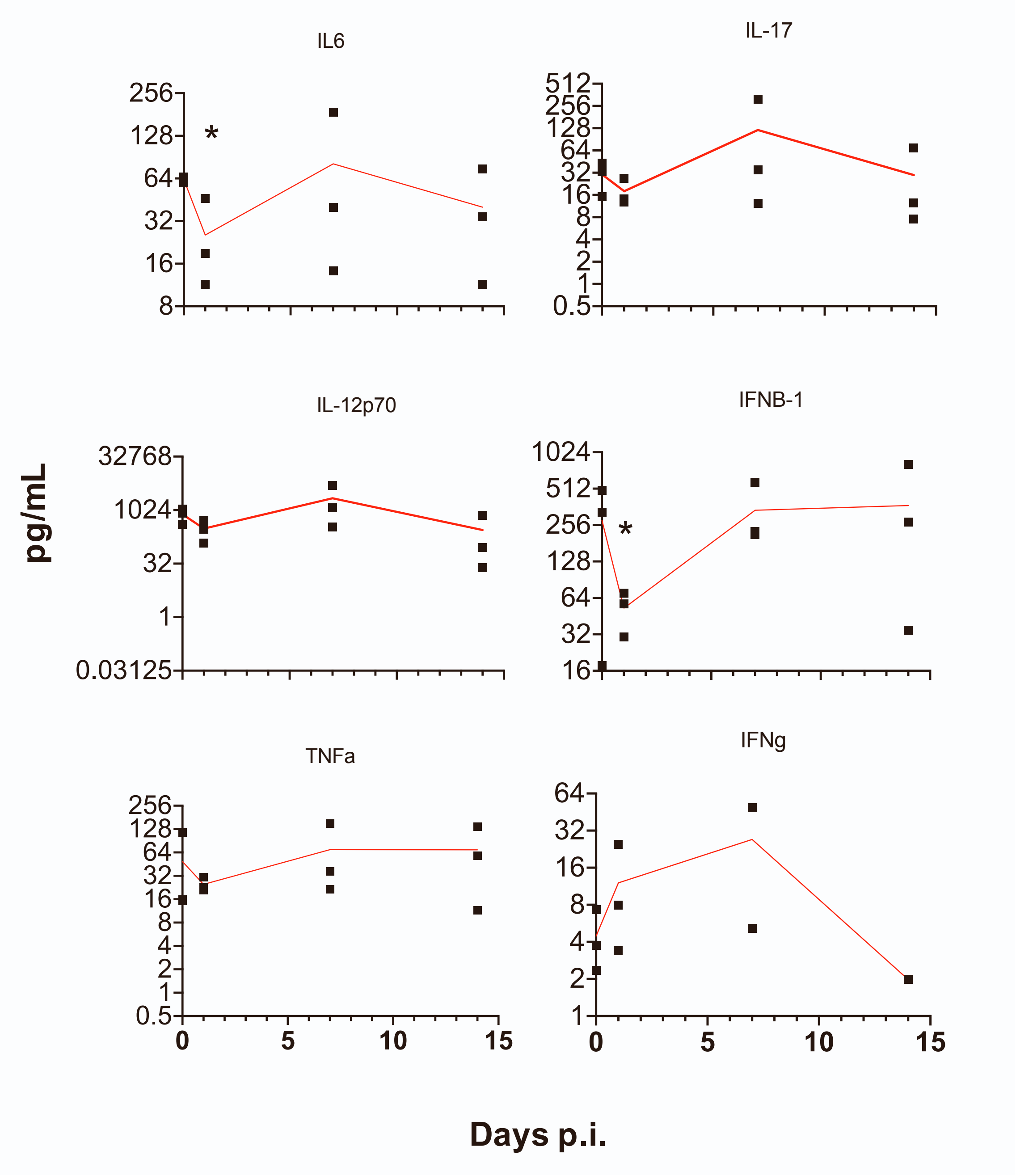

Supplementary Figure 1: Serum Cytokine Expression after Ad-SPC-Cre

Selected cytokines from Luminex cytokine panel, assessed for concentration in serum at 24 hours, 7 days, or 14 days p.i. with 2.5 x 10^7 PFU Adeno-SPC-Cre. n=3 mice per timepoint. Data points not shown are outside the range of detection of the assay (0pg/mL). * indicates p<0.05 unpaired T test vs uninfected. Related to **Figure 2C**.

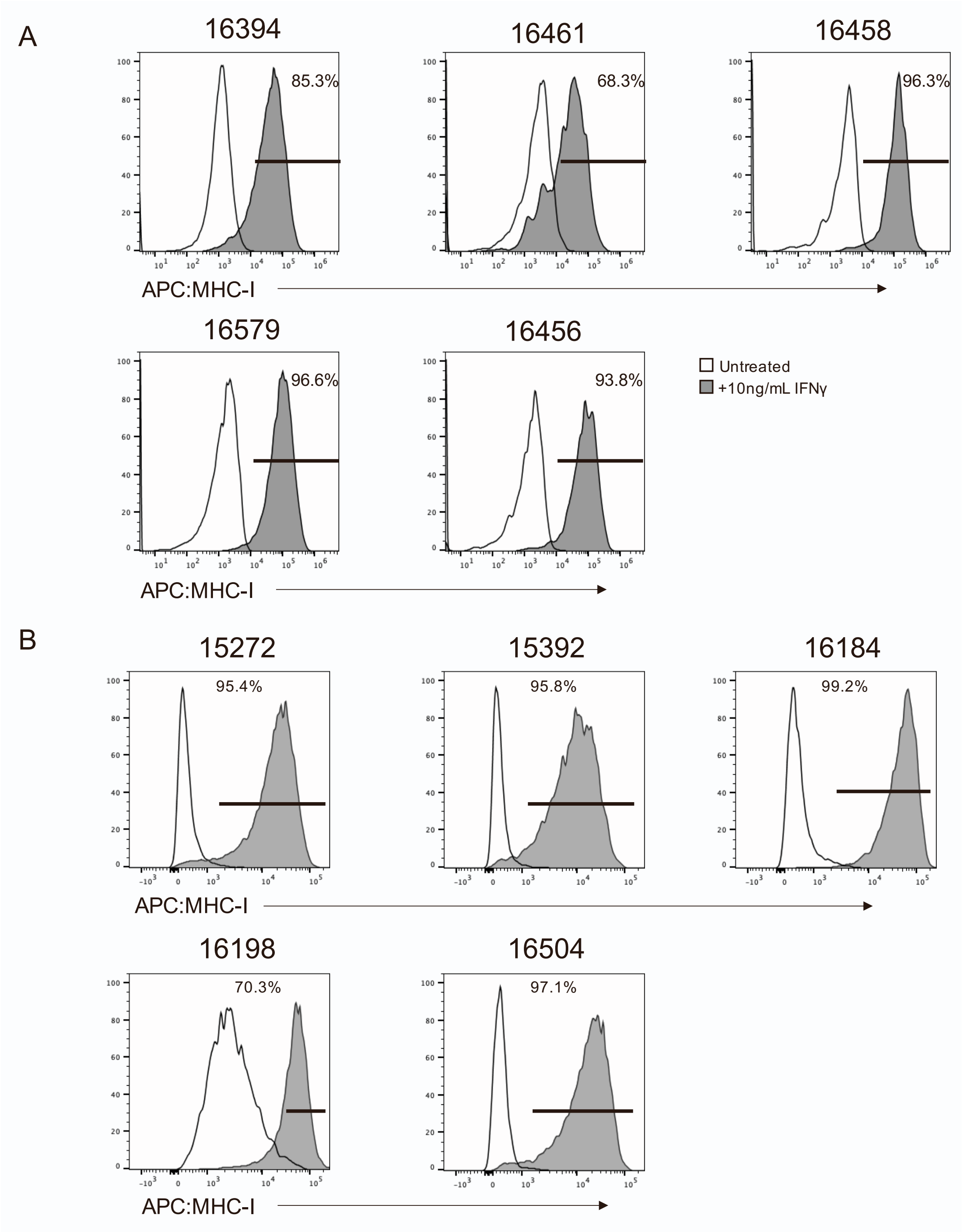

Supplementary Figure 2: KP-NINJA + Cre LV cell lines upregulate MHC-I after stimulation with IFNγ

Untreated vs interferon gamma-stimulated treated primary lung cell lines, pregated on GFP+ cells. a, KP-NINJA cell lines. b, LucOS cell lines. Related to **Figure 3F**.

aacATGGTGAGCAAGGGCGAGGAGCTGTTCACCGGGGTGGTGCCCATCCTGGTCGAGCTGGACGGCGACG
TAAACGGCCACAAGTTCAGCGTCCGCGGCGAGGGCGAGGGCGATGCCACCAACGGCAAGCTGACCCTGA
AGTTCATCTGCACCACCGGCAAGCTGCCCGTGCCCTGGCCCACCCTCGTGACCACCTTCGGCTACGGCGT
GGCCTGCTTCAGCCGCTACCCCGACCACATGAAGCAGCACGACTTCTTCAAGTCCGCCATGCCCGAAGGCT
ACGTCCAGGAGCGCACCATCTCTTTCAAGGACGACGGTACCTACAAGACCCGCGCCGAGGTGAAGTTCGA
GGGCGACACCCTGGTGAACCGCATCGAGCTGAAGGGCATCGACTTCAAGGAGGACGGCAACATCCTGGG
GCACAAGCTGGAGTACAACTTCAACAGCCACTACGTCTATATCACGGCTGGATCTGCAGGATCAGCGGCCG
GCTCAGGCGAGTTTAAAGCTGTGTACAACTTTGCAACATGCGGGATCGACTACAAGGACGACGATGACAAG
GGTTTGAACGGCCCTGACATTTACAAGGGAGTTTATCAGTTTAAATCCGTTGAGTTTGACGGCAGCGCGGGC
AGTGCTGCTGGGTCAGGAGAATTCGACAAGCAGAAGAACTGCATCAAGGCTAACTTCAAGATCCGCCACAA
CGTTGAGGACGGCAGCGTGCAGCTCGCCGACCACTACCAGCAGAACACCCCCATCGGCGACGGCCCCGT
GCTGCTGCCCGACAACCACTACCTGAGCCATCAGTCCAAGCTGAGCAAAGACCCCAACGAGAAGCGCGAT
CACATGGTCCTGCTGGAGTTCGTGACCGCCGCCGGGATTACACATGGCATGGACGAGCTGTACAAGT

GP33-41:

TTAAAGCTGTGTACAACTTTGCAACATGCG

FLAG tag:

GACTACAAGGACGACGATGACAAG

GP 61-80:

GGTTTGAACGGCCCTGACATTTACAAGGGAGTTTATCAGTTTAAATCCGTTGAGTTT

Supplementary Figure 3: Sequence of the Cre-GFP33 LV neoantigen lentiviral vector. Related to **Figure 3G**.

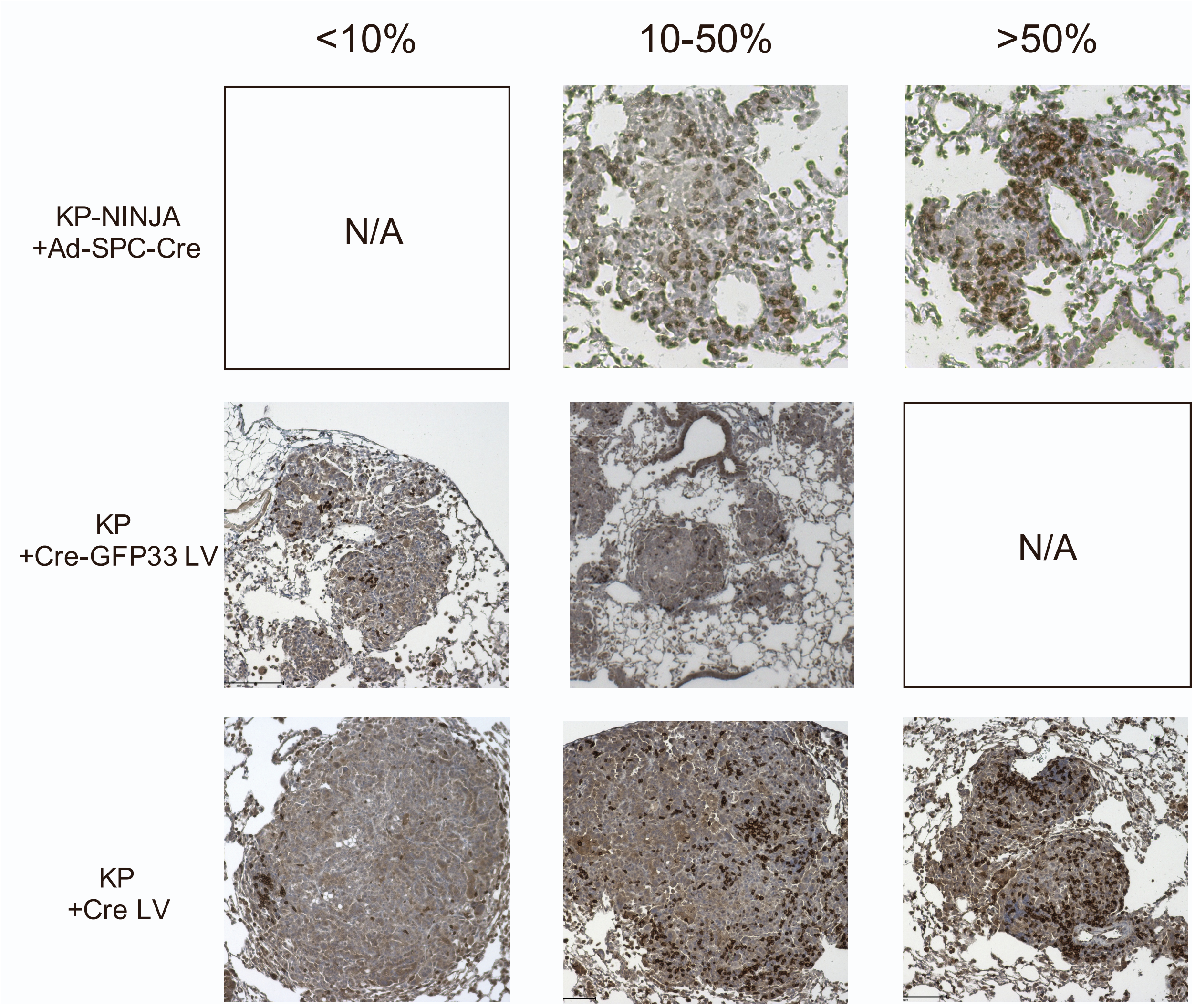

Supplementary Figure 4: T cell infiltration in lung tumors

Representative aCD3 IHC for each blinded infiltration score category, for each tumor model: KP-NINJA + Ad-SPC-Cre, KP + Cre-GFP33 LV, and KP + Cre LV. N/A indicates no samples received that score. Related to **Figure 4A**.

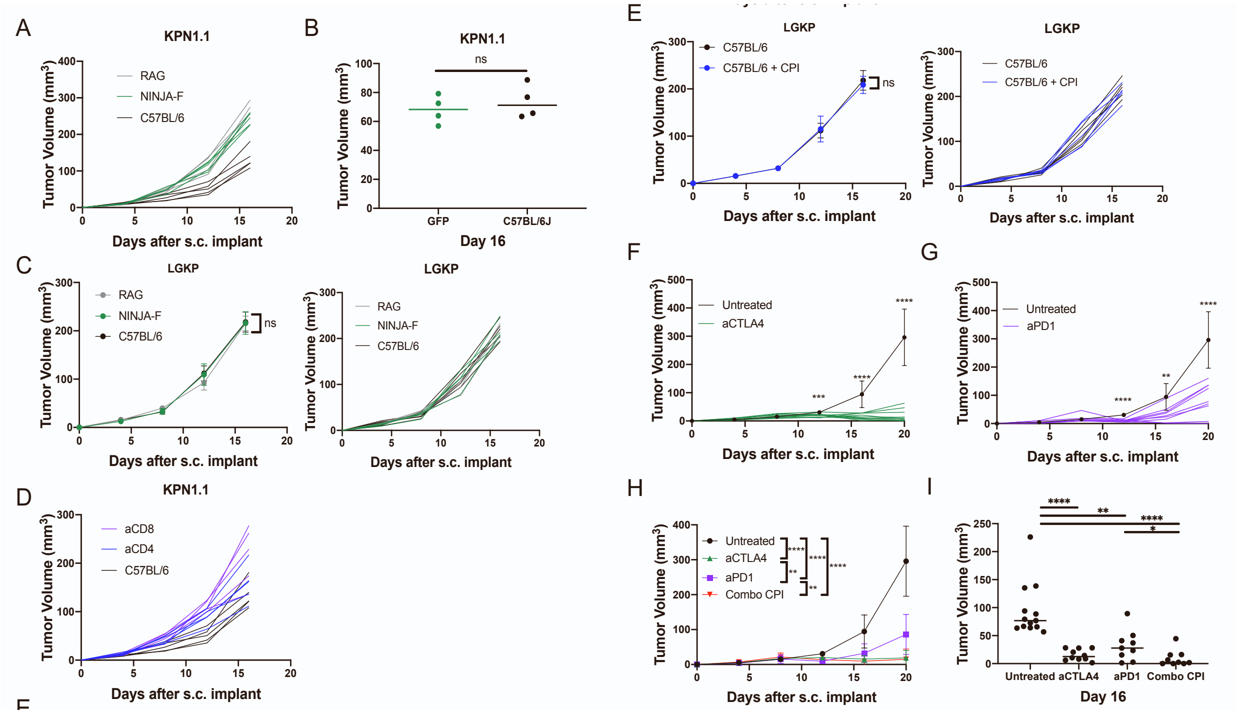

Supplementary Figure 5: KPN1.1 and KP Control Cell Line Transplant Tumors

a, Individual KPN1.1 tumor growth curves, related to **Figure 5C**.

b, Growth at Day 16 for KPN1.1 in C57BL/6J and Il4GFP (4get) GFP tolerant recipients. Not significant by unpaired T Test. N=4, single experiment.

c, Growth of non-antigen expressing primary KP lung tumor cell line (LGKP) control, related to **Figure 5C**. Individual tumor growth curves right panel, averages left panel. ns = not significant by Two-way ANOVA.

d, Individual KPN1.1 tumor growth curves, related to **Figure 5D**.

e, Growth of non-antigen expressing primary KP lung tumor cell line (LGKP) control in experiment shown in 5G-H in main text. Individual tumor growth curves right panel, averages left panel. ns = not significant by Two-way ANOVA. N=4-5 per group

f, Growth of KPN1.1 with or without anti-CTLA4 200ug dosing on days 3,6,9. Average of untreated group (error bars = SD, n=13, from three experiments, also shown in **g**), individual tumor growth curves for treatment group (n=10, from three experiments).

g, Growth of KPN1.1 with or without anti-PD1 200ug dosing on days 3,6,9. Average of untreated group (error bars = SD, n=13, also shown in **f**), individual tumor growth curves for treatment group (n=9). (** = p<0.005, *** = p<0.0005 **** = p<0.0001 unpaired T Test.)

h, Average tumor growth for all conditions in same experiment used in **f** and **g,** (significance comparison is shown for day 20; ** = p<0.005, **** = p<0.0001unpaired T Test)

i, Day 16 timepoints for tumor growth in same experiment as **f** and **g**. (* = p<0.05, ** = p<0.005, **** = p<0.0001 unpaired T Test.)

e-i, Related to **Figure 5G-H.**

Tumor Cell Antibody Panel	Source	Identifier	Dilution
EpCam/CD326-PeCy7 (clone: G8.8)	eBioscience	Cat# 118216	1:500
CD45-Pacific Blue (clone:30-F11)	BioLegend	Cat# 103125	1:200
CD31-BV605 (clone:390)	BioLegend	Cat# 104247	1:300

T Cell Antibody Panels	Source	Identifier	Dilution	Panel
CD45-PECF594 (clone:30-F11)	BD Biosciences	Cat# 562420	1:200	1,2
CD8-PECy5 (clone:CT-CD8a)	Thermo Fisher Scientific	Cat# MA5-17601	1:500	1,2
Thy1.2-BV605 (clone:30-H12)	BioLegend	Cat# 105343	1:500	1,2,3
Tim3-PECy7 (clone:RMT3-23)	BioLegend	Cat# 119716	1:500	1,2,3
PD-1-BV421 (clone:29F.1A12)	BioLegend	Cat# 135217	1:500	1,2,3
SLAMF6-PE (clone:330-AJ)	BioLegend	Cat# 134606	1:500	1,2
TCF1-AF488 (clone: 812145)	Fisher Scientific	Cat# IC8224G	1:100	1,2,3
CD44-BV711 (clone:IM7)	BD Biosciences	Cat# 563971	1:500	2
CX3CR1-BV711 (clone: SA011F11)	Biolegend	Cat# 149031	1:500	1
CD4-PerCP (Clone RM4-5)	BioLegend	Cat# 100538	1:500	3
CD44-APC-Cy7 (Clone IM7)	BioLegend	Cat# 103027	1:500	3
SlamF6- BV750 (Clone 13G3)	BD Biosciences	Cat# 747169	1:800	3
Granzyme B- PE (Clone GB11)	BD Biosciences	Cat# 561142	1:100	3
CD127-PE Dazzle (Clone A7R34)	BioLegend	Cat# 135032	1:200	3
CD8a-BUV395 (Clone 53-6.7)	BD Biosciences	Cat# 565968	1:500	3

In Vitro MHC-I/PDL1 Antibody Panel	Source	Identifier	Dilution
MHC Class I (clone: H2-kB)	eBioscience	Cat# 17-5958-82	1:400
PD1-PE (clone:10F.9G2)	BioLegend	Cat# 124308	1:400

Supplementary Table 1: Selected Antibody Panels. Related to STAR methods.
